# Models for Access to Maternal Smoking cessation Support (MAMSS): a study protocol of a quasi-experiment to increase the engagement of pregnant women who smoke in NHS Stop Smoking Services

**DOI:** 10.1186/1471-2458-14-1041

**Published:** 2014-10-06

**Authors:** Lorna Bennett, Aimee Grant, Siobhan Jones, Mererid Bowley, Christian Heathcote-Elliott, Catrin Ford, Angela Jones, Rachel Lewis, Margaret Munkley, Carol Owen, Annie Petherick, Shantini Paranjothy

**Affiliations:** Public Health Wales NHS Trust, Temple of Peace and Health, Cathay’s Park, Cardiff, CF10 3NW UK; Cochrane Institute of Primary Care and Public Health, Cardiff University School of Medicine, 5th Floor Neuadd Meirionnydd, Heath Park, Cardiff, CF14 4YS UK; Public Health Wales NHS Trust (Betsi Cadwaladr University Health Board local public health team), 10 Llys Castan, Ffordd y Parc, Parc Menai, Bangor, Gwynedd LL57 4DF UK; Public Health Wales NHS Trust (Aneurin Bevan University Health Board local public health team), Llanarth House, Newbridge Gateway, Bridge Street, Newbridge, NP11 5GH UK; Public Health Wales NHS Trust (Abertawe Bro Morgannwg University Health Board local public health team), Floor 12, The Oldway Centre, 36 Orchard Street, Swansea, SA1 5AW UK; Public Health Wales NHS Trust (Cwm Taf University Health Board local public health team), Keir Hardie Health Park, Aberdare Road, Merthyr Tydfil, CF48 1BZ UK; Public Health Wales Trust (Betsi Cadwaladr University Health Board local public health team), Preswylfa, Hendy Road, Mold, Flintshire, CH7 1PZ UK; Public Health Wales NHS Trust, 14 Cathedral Road, Cardiff, CF11 9LJ UK

**Keywords:** Smoking, Smoking cessation, Stop smoking services, Pregnant women, Pregnancy, Midwife, Maternity support worker, Quasi-experiment, Service delivery

## Abstract

**Background:**

Maternal smoking is a key cause of poor outcomes for mothers, babies and children and Wales has higher rates of smoking in pregnancy than any other UK country. Despite various improvements within the NHS Stop Smoking Service to strengthen the intervention for pregnant women, referrals and successful quit attempts for this group have continued to remain extremely low. A key element of UK national guidance for smoking cessation during pregnancy is to provide a flexible and tailored service to help increase levels of engagement. This study aims to test the effectiveness of three different models of service delivery to address the gap in the evidence base about how to deliver a flexible, tailored smoking cessation service to pregnant women.

**Methods:**

This study will adopt a quasi-experimental design over a 12 month period. The setting is four of Wales’ seven Health Boards using an integrated approach between maternity services, local public health teams and the NHS Stop Smoking Service. Core recommendations from UK public health guidance are being implemented across intervention and usual care sites. Stop smoking support for pregnant women in intervention sites is being delivered more flexibly than in usual care sites. Both qualitative and quantitative approaches will be adopted to capture important contextual information and consider multiple perspectives. A health economic analysis will be undertaken using a cost-consequences analysis approach. The primary outcome measure is engagement with stop smoking services (defined as having at least one face-to-face therapeutic contact with a clinician).

**Discussion:**

Supporting pregnant women to stop smoking is a challenging area of public health. The proposed study will address several areas where there are key evidence gaps relating to smoking cessation interventions for pregnant women. Specifically, how best to encourage pregnant women to attend a specialist stop smoking support service, how to deliver the service and who should provide it.

**Electronic supplementary material:**

The online version of this article (doi:10.1186/1471-2458-14-1041) contains supplementary material, which is available to authorized users.

## Background

A third of pregnant women living in Wales smoke before or during their pregnancy compared to just over a quarter of women living in other areas of the UK [1]. This is a major concern since maternal smoking is a key cause of poor outcomes for mothers, babies and children. It is associated with increased risk of miscarriage, perinatal death, prematurity, low birth weight and congenital abnormalities in the baby in particular of the heart, face and limbs [2, 3]. Supporting pregnant women to stop smoking is thus an important area of public health.

High quality evidence exists for interventions to promote smoking cessation in pregnancy. A Cochrane review found that psychosocial interventions led to a reduction in smoking during pregnancy and reduced the risk of low birth weight and pre-term birth [4]. UK public health guidance on interventions aimed at stopping smoking in pregnancy and following childbirth provides a clear pathway and referral mechanism for pregnant smokers into NHS Stop Smoking Services [5].

A key component of this guidance is carbon monoxide (CO) testing for all pregnant women to assess their level of exposure to CO either as smokers themselves or from other sources. Test results are used to ensure the appropriate identification of smokers for onward referral to specialist support services. It has been estimated that 20 per cent of smokers deny their habit when cotinine measurements are compared with self-report [6], leaving these pregnant smokers without the information available to facilitate a quit attempt. It has been reported in UK based research that employing the carbon monoxide test in conjunction with self-report improves the identification of smokers, but that maternity staff have found it difficult to discuss smoking and administer the test [7].

A further key element of UK national guidance for smoking cessation during pregnancy is to provide a flexible and tailored service for pregnant women to help increase levels of engagement. This is due to the fact that pregnant women may not feel comfortable or have all their needs met when attending generic services. Many English stop smoking services offer a flexible service for pregnant women [8]. However, it is not known how best to deliver such a service or who should provide it.

Within the Welsh NHS Stop Smoking Service^a^, various service improvements have occurred since 2009 with a view to strengthening the referral system for pregnant women who smoke from antenatal units to the stop smoking service. These service improvements have included additional training of midwifery staff, in line with National Institute for Health and Care Excellence (NICE) guidance, to encourage the use of CO testing. Despite this, referrals into the service and engagement with pregnant women has continued to remain extremely low. For example, data from the Stop Smoking Service show that in the referral period May 2012-March 2013, 1,817 pregnant smokers were referred to the service from maternity services, but only 529 went on to accept an appointment. At this time, there were around 32,000 births in Wales [9] based on a smoking prevalence of 33% [1], 5.6% of smokers were referred to the service, and 1.6% of smokers accepted an appointment. In response, key partners were brought together across Wales to develop a public health study, Models for Access to Maternal Smoking cessation Support (MAMSS) with the aim of testing the effectiveness of three different models of service delivery to address the gap in the evidence base about how to deliver a flexible tailored smoking cessation service to pregnant women.

## Methods

This study will be undertaken as a quasi-experimental design. Donabedian’s conceptual model for examining health services and evaluating quality of care as a framework for examining structures, processes and outcomes will be applied [10]. The setting will be four of Wales’ seven Health Boards. These are single local health organisations responsible for planning, securing and delivering all healthcare services within a geographical area. The Health Boards involved in the study are self-selected and are geographically diverse. Total population figures range from 294,497 to 690,434 [11] and live births range from 63.9 to 66.3 per 1000 women aged 15-44 [9] (see Table 1).

**Table 1:** **Total population and live births per 1000 for Health Boards participating in MAMSS**

Health board	Total population [11]	Live births per 1000 women aged 15-44 [9]
Health board 1	519,781	60.0
Health board 2	577,981	61.8
Health board 3	690,704	64.8
Health board 4	294,497	61.7

### Interventions

**Figure 1:**
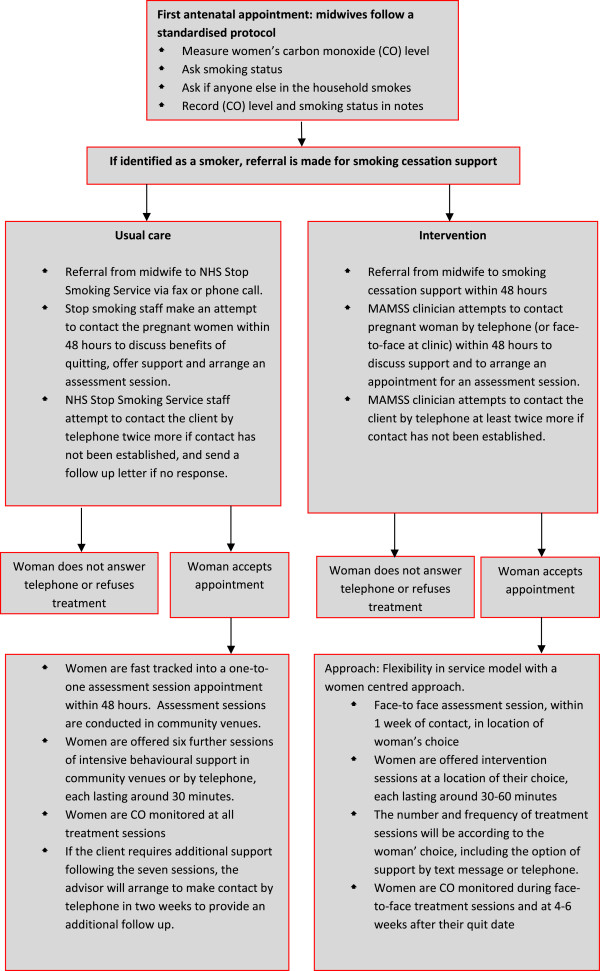
**Referral and treatment in usual care and intervention sites.**

**Figure 2:**
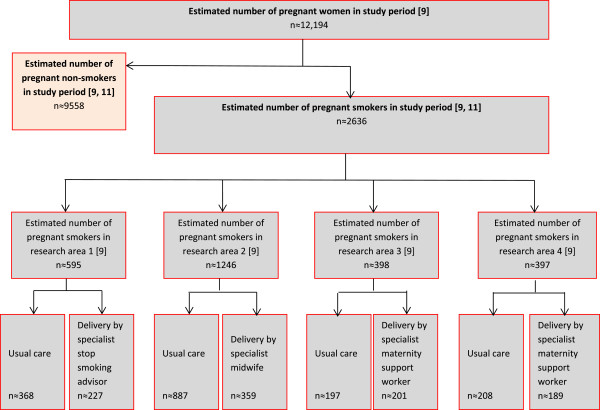
**Recruitment flow diagram.**

The study will take place over 12 months using an integrated approach between maternity services, local public health teams and the NHS Stop Smoking Service. In each Health Board, an intervention and a usual care site will be selected from existing community midwifery teams. Sites will be selected on the basis of high rates of pregnant smokers, and a similar demographic profile in intervention and usual care sites within each Health Board. Pregnant smokers (i.e. those who are self-reported current smokers or have a CO reading # 7 ppm^b,^^c^ or who have quit in the two weeks prior to their booking appointment [5]) will be referred to the intervention or usual care NHS Stop Smoking Service on an opt out basis (see Figures 1 and 2). This automatically refers pregnant smokers to the service. The two main points for referral are the first antenatal appointment (at home or in a community antenatal clinic by the midwife) or subsequent scheduled antenatal care visits (by the midwife). However, the referral process can take place at any stage during pregnancy, for example, during the postpartum period whilst under the care of a midwife.

In intervention sites, pregnant smokers will be referred to one of three different models of service delivery where smoking cessation services are provided by maternity support workers, midwives or smoking cessation advisors dedicated to working with pregnant women, all offering a flexible service designed to meet the needs of the women. The core elements of the evidence base to be implemented in all intervention sites will include:

-  Strict adherence to NICE opt out smoking cessation pathway for pregnant women, including CO monitoring.
-  Smoking cessation services being more closely aligned to maternity services (provided as part of the package of maternity care).
-  Referral from midwife to smoking cessation support within 48 hours
-  Flexibility in service model with a women centred approach.

Each intervention site will recruit a whole-time equivalent (WTE) maternity support worker (employed by the Health Board), midwife (employed by the Health Board) or dedicated Stop Smoking Advisor for pregnant women (employed by the NHS Stop Smoking Service) who will deliver stop smoking support to pregnant women. These staff will receive referrals directly from midwives to provide an intensive smoking cessation intervention at times and settings of the women’s choice, including home visits. During every subsequent antenatal appointment, women will be asked about their smoking status and referred as appropriate. If a woman has declined treatment (i.e. she opts-out by not answering the telephone to stop smoking services or refuses an assessment appointment) she will be asked if she would like to be re-referred during each follow-up appointment.

Clients in intervention sites will be offered smoking cessation support for the duration of their pregnancy if required. The support they receive will follow an adapted behavioural intervention model documented in the NHS Stop Smoking Service delivery manual, which is based on withdrawal oriented therapy (Maudsley model) [12]. This may involve a combination of telephone and face to face support as well as motivational support by email and or text, dependent upon the needs of the client (see Figure 1). A detailed manual for specialist smoking cessation staff has been developed for use in all intervention sites detailing the referral pathway, intervention programme, policies, procedures and support materials.

### Comparison group (usual care)

Pregnant smokers will be referred on an opt out basis to NHS Stop Smoking Services in usual care sites. The core elements of this service are:

-  Referrals received by NHS Stop Smoking Service from midwives via fax or phone call.
-  Stop smoking staff make an attempt to contact the pregnant women within 48 hours to discuss benefits of quitting, offer support and arrange an assessment session.
-  NHS Stop Smoking Service staff attempt to contact the client by telephone twice more if contact has not been established, and send a follow up letter if no response.
-  Clients who do not opt out are fast tracked into a one-to-one assessment session appointment within 48 hours. Assessment sessions are conducted in community venues.
-  Women are offered six further sessions of intensive behavioural support in community venues or by telephone.
-  If the client requires additional support following the seven sessions, the advisor will arrange to make contact by telephone in two weeks to provide an additional follow up.

### Outcome measures

The primary outcome measure is engagement with stop smoking services (defined as having at least one face-to-face therapeutic contact with an advisor) and secondary outcome measures are pregnant smokers who set a quit date; pregnant smokers who quit at four weeks follow-up (CO verified); smoking status at the time of birth or third trimester and 52 weeks; and birth outcomes (low birth weight (<2500 g), preterm birth <37 weeks).

Process measures will assess fidelity to protocols, feasibility, acceptability, maintenance and sustainability should the interventions be rolled out on a wider scale. Data will be obtained from available data sources within maternity services and the NHS Stop Smoking Service on specific aspects of the referral pathway and treatment programme.

### Sample

Women who are pregnant and smoke or who have quit smoking in the two weeks prior to their initial antenatal booking appointment will be eligible to participate. The inclusion criteria are pregnant or postnatal women, under the care of a midwife, who are either a current smoker (self-report or verified through CO reading of ≥ 7 ppm^2^) or ex-smoker (quit within two weeks prior to booking appointment). The exclusion criteria are pregnant women who are not smokers or who have reduced mental capacity.

### Sample size

We hypothesise that the flexible models of service delivery can increase the percentage of pregnant women who engage with smoking cessation services from 10 per cent (current rate of engagement in NHS England smoking cessation services) to 25 per cent. A sample size of 146 pregnant smokers in each group within each Health Board will allow a 15 per cent difference to be detected in the proportion of pregnant smokers who engage, with a five per cent type I error rate and 90 per cent power.

### Quantitative data collection

Quantitative data collection will principally utilise routine data from: i) the Health Board’s electronic Patient Administration System (PAS) and Maternity Information System, which contain information about expectant mothers smoking status and birth outcomes and ii) the NHS Stop Smoking Service database which will record referral and treatment information for all intervention and usual care clients. Each Health Board uses a national standardised maternity record. The maternity record aims to improve quality and safety of maternity care and is updated regularly according to national guidelines to represent the latest recommendations for best practice. Within the record, there is a comprehensive risk assessment which identifies individual risk factors, including smoking status, for the expectant mother. Data from the patient hand-held record is entered into each Health Board’s electronic PAS and Maternity Information System (where available). Additional file 1 provides detail about the specific data items to be extracted from the PAS and Maternity Information System for this study.

Information about the referral pathway and stop smoking treatment programme will be entered onto a separate database used by the existing NHS Stop Smoking Service which has recently undergone developments including the ability to conduct e-referrals. Data items to be extracted are detailed in Additional file 1. To facilitate this process, Memoranda of Understanding (MoU) will be established between Public Health Wales Informatics team and the four participating Health Boards and between Public Health Wales Informatics and the NHS Stop Smoking Service which will allow Public Health Wales to hold and process Health Board and NHS Stop Smoking Service data on their behalf. Approval will be sought from each Health Board and the NHS Stop Smoking Service to allow Public Health Wales Informatics to release anonymised data to specified analysts for use in accordance with an agreed analysis plan. The Public Health Wales Informatics team will act as the NHS Stop Smoking Service’s and the Health Board’s agent in this process and undertake any linking that has to be undertaken before anonymising the data and providing it to analysts. All data will be anonymised, therefore patient confidentiality will be maintained. All documents will be stored securely and only accessible by study staff and authorised personnel. The study will comply with the Data Protection Act which requires data to be anonymised as soon as it is practical to do so.

### Analysis

Baseline demographic and social characteristics of pregnant smokers in both intervention and usual care sites will be summarised using descriptive statistics. Comparisons between the two groups will be carried out using a Student’s *t*-test or a non-parametric test for continuous data and a chi-squared test or a Fisher’s exact test for categorical data. Characteristics of women who do not opt out of the referral will be compared to those who do. Continuous data will be expressed as mean values with standard deviations and categorical data will be presented as counts with percentages. A single logistic regression model will be developed to obtain odds ratios for the comparison of different interventions with usual care, and for comparisons between interventions. Differences in baseline characteristics of individuals and referral rates for Stop Smoking Services will be adjusted for. To account for the geographical clustering of individuals, robust standard errors will be calculated.

### Qualitative evaluation

Staff delivering treatment or referring women for treatment in all usual care areas and in three of the intervention areas will be invited to take part in semi-structured interviews. All 32 usual care advisors will be emailed with an information sheet and asked to contact the researcher if they are experienced in supporting pregnant women. If low response occurs, further invitations will be sent by email. Staff in selected intervention areas will be recruited via the Health Board’s lead or co-lead for the study. Staff will have the opportunity to undertake an interview either face-to-face within a private room in their work place, or by telephone at a time that is convenient for them.

Clients who receive treatment will be invited to take part in the study by the staff delivering their treatment. If a client expresses an interest in the study, staff will provide them with an information sheet, containing contact details for the researcher. The client can then opt in by contacting the researcher. Whilst staff introduction will introduce bias into the sample, and a low response rate is likely due to the opt-in procedure, it will allow vulnerable women the opportunity to take part in research. In order to minimise data collection costs in a large research site, ensure researcher safety and to encourage clients to be open about their treatment, interviews will be conducted by telephone. Whilst traditionally face-to-face interviewing has been seen as important for health research, the advantages of telephone interviewing are increasingly recognised [13].

If participants consent, interviews will be audio recorded and transcribed verbatim. Comprehensive notes will be taken if consent is refused. Qualitative data will be analysed using a structured approach, Framework analysis, which involves five stages: familiarisation, constructing an initial framework, coding, reviewing data extracts and conclusion drawing [14]. Transcripts will be coded by hand, before being displayed in tables, with all instances of one code examined together. Finally conclusions will be drawn by identifying salient themes in the tables. The use of a structured approach aims to minimise researcher bias. Moreover, each stage of the analysis will be agreed by two researchers, with inconsistencies discussed and resolved.

### Economic evaluation

A health economic analysis will be undertaken using a cost-consequences analysis approach. Over a period of 12-months, cost data will be collected on wages, travel, training, NRT prescribing and average length of intervention per client. Summary information for these costs will be presented in a tabular format against the consequences of each intervention. Consequences are defined as the primary and secondary outcome measures previously described.

### Ethical considerations

We contacted the Research and Development manager for all four sites. One Health Board, Cwm Taf Health Board, received NHS ethical approval (South East Wales Research Ethics Committee). In the other three sites the research was declared service evaluation by the Research and Development manager (Abertawe Bro Morgannwg University Health Board ), the Clinical Audit and Effectiveness Manager (Betsi Cadwaladar University Health Board) and the Aneurin Bevan Health Board Research Risk Review Committee (Aneurin Bevan Health Board). As a result the project did not require NHS ethical approval. This decision was approved by the NHS Research Ethics Committee (REC) for Wales. Research and development approvals were obtained from all four Health Boards and Public Health Wales NHS Trust.

## Discussion

A quasi-experimental design for the proposed study was adopted as it was not logistically feasible to conduct a cluster randomised controlled trial in the desired timescales and within the available budget [15]. Although the randomised controlled trial is generally considered to have the highest level of credibility with regard to assessing causality, the use of a comparison group helps prevent certain threats to validity including the ability to statistically adjust for confounding variables.

The proposed study will address several areas where there are key evidence gaps relating to pregnant women and smoking cessation interventions. Specifically, how best to encourage pregnant women to attend a specialist stop smoking support service, how to deliver the service and who should provide it. The study will reveal important findings in relation to service delivery and enable local services to improve their effectiveness in a challenging area of public health. Ensuring better identification of pregnant smokers and providing access to timely support from smoking cessation specialists is crucial to reducing the number of women smoking during pregnancy in Wales. Investigating ways to provide a more flexible, women-centred approach is an important development which will assist in reducing the range of adverse factors for both mother and baby associated with maternal smoking behaviour.

## Endnotes

^a^The NHS Stop Smoking Service in Wales is known as Stop Smoking Wales. The service is funded and run by Public Health Wales NHS Trust - an NHS organisation providing professionally independent public health advice and services to protect and improve the health and wellbeing of the population of Wales. It contributes to national and local tobacco control initiatives and has a key role in reducing the impact of tobacco on the health of people in Wales.

^b^A lower level (e.g. 3 ppm) may apply for light/infrequent smokers. A higher level might apply if prior exposure to other sources of pollution e.g. traffic fume, leaky gas appliances [5].

^c^The use of 7 ppm^2^ was adopted as it was accepted best practice, contained in NICE guidance, at the time of the research design. It is likely that NICE will move to 4 ppm^2^ in the future, and some existing research is already using this definition. We recommend that future research use a definition of 4 ppm^2^.

## Electronic supplementary material

Additional file 1: **Data items to be extracted from NHS systems.** (PDF 204 KB)

## Abbreviations

CO: Carbon monoxide
MAMSS: Models for access to smoking cessation support
MoU: Memorandum of understanding
NHS: National Health Service
NICE: National Institute for Health and Care Excellence
NRT: Nicotine replacement therapy
PAS: Patient administration system
UK: United Kingdom
WTE: Whole time equivalent.
