# Supplementary material for: Models for Access to Maternal Smoking cessation Support (MAMSS): a study protocol of a quasi-experiment to increase the engagement of pregnant women who smoke in NHS Stop Smoking Services
Source: BMC Public Health. 2014 Oct 6;14:1041. doi: 10.1186/1471-2458-14-1041 (PMC4287561; doi:10.1186/1471-2458-14-1041)
Supplement: Supplementary file 1 — Additional file 1: Data items to be extracted from NHS systems. (PDF 204 KB) [file 12889_2013_7355_MOESM1_ESM.pdf]

Models for Access to Maternal Smoking cessation Support (MAMSS): a study protocol to develop an intervention to increase the engagement of pregnant women who smoke in NHS Stop Smoking Services

Additional files

1. Data items to be extracted from local Patient Administration System

NHS number
Age (at first maternity appointment)
Postcode
Agreed estimated date of delivery
Employment
Occupation
Do you smoke?
If yes, how many cigarettes do you smoke each day?
Have you smoked in the last 2
Treatment sessions? When did you
stop smoking?
Have you been offered advice on how to stop smoking?
Have risks of smoking been explained to you?
Have you received written information on how to stop smoking?
CO reading
Date of referral to stop smoking services
Does your current partner smoke?
Are you exposed to cigarette smoke at home?
Which groups do you most identify with? (Ethnicity)
Date and time of birth
Live / still birth
Birth weight
Smoking status at birth
Gestational age

2. Data items to be extracted from NHS stop smoking service database regarding referral

NHS number
Date referral received
Name of midwife
Hospital
Local Area
Smoker?
If ex-smoker date when stopped
For smokers only, permission to pass details to Stop Smoking
Service
Name
Address
Postcode
Contact telephone number
Mobile telephone number
Due date
CO Reading
Attempt to contact by telephone 1
Attempt to contact by telephone 2
Attempt to contact by telephone 3 (if unsuccessful, letter to be sent)
Letter Sent
Accept/reject assessment session appointment
Client Code
Date & venue of assessment session appointment
Advisor

3. Data items to be extracted from NHS stop smoking service database regarding treatment

Demographics
NHS Number
Patient ID
Name
Gender
Address
Postcode
Telephone number (home, work, mobile)
Email address
Date of birth
Age started smoking
Age at time of treatment
Age at quit date
Ethnicity
Occupation
Deceased
Used service in past year
Local Health Board
General Practitioner
General Practice
How heard of service

Specialist support needed?
Pregnant?
If pregnant, due date
Breastfeeding
Pre-operative
If pre-operative, operation date and hospital

Consent
General Practice contact consent
Monitoring consent
Follow up consent
Data protection statement signed

Smoking history and nicotine addiction
Previous number of quit attempts
Previous NRT use
Previous Zyban (bupropion hydrochloride) use
Previous Champix (Varenicline) use
How soon after waking is first cigarette smoked?
Do you find it difficult to refrain from smoking where it is forbidden?
Which cigarette would you find it most difficult to give up?
Number of cigarettes smoked per day
Do you smoke more within first few hours of waking?
Do you smoke when ill in bed?
Nicotine addiction (Fagerstrom score)

Treatment
Initial contact date
Appointment within four weeks of initial contact
Assessment questionnaire return date
Advisor (treating clinician)
Venue
Venue type

Detailed treatment record
Assessment session date
Assessment session attendance
Assessment session CO reading
Treatment session 1 date
Treatment session 1 attendance
Treatment session 1 CO test attempted
Treatment session 1 CO reading
Treatment session 2 date
Treatment session 2 attendance
Treatment session 2 CO test attempted
Treatment session 2 CO reading
Treatment session 3 date
Treatment session 3 attendance
Treatment session 3 CO test attempted
Treatment session 3 CO reading
Treatment session 4 date
Treatment session 4 attendance
Treatment session 4 CO test attempted
Treatment session 4 CO reading
Treatment session 5 date
Treatment session 5 attendance
Treatment session 5 CO test attempted
Treatment session 5 CO reading
Treatment session 6 date
Treatment session 6 attendance
Treatment session 6 CO test attempted
Treatment session 6 CO reading
General practitioner pharmacotherapy letter given?
Pharmacy pharmacotherapy letter given?

Outcome measures
Attended treatment session
Set quit date
Treated smoker
4 week follow up completed
4 week follow up completion date
4 week self-reported quit status
4 week CO test reading
CO reading confirms self-reported 4 week quit status
52 week follow up completed
52 week self-reported quit status
